# Small molecular weight epigenetic inhibitors modulate the extracellular matrix during pancreatic acinar ductal metaplasia

**DOI:** 10.1016/j.bbrc.2024.150496

**Published:** 2024-08-03

**Authors:** Corey M. Perkins, Yating Mao, Jinmai Jiang, Diana J. Wilkie, Bo Han, Qi-Yin Chen, Hendrik Luesch, Jamel Ali, Thomas D. Schmittgen

**Affiliations:** a Department of Pharmaceutics, College of Pharmacy, University of Florida, Gainesville, FL, USA; b Department of Chemical and Biomedical Engineering, FAMU-FSU College of Engineering, Tallahassee, FL, USA; c National High Magnetic Field Laboratory, Tallahassee, FL, USA; d Department of Behavioral Nursing Science, College of Nursing, University of Florida, Gainesville, FL, USA; e Department of Surgery, University of Southern California, Los Angeles, CA, USA; f Department of Medicinal Chemistry, College of Pharmacy, University of Florida, Gainesville, FL, USA; g Center for Natural Products, Drug Discovery and Development, College of Pharmacy, University of Florida, Gainesville, FL, USA; h Florida-California Cancer Research Education and Engagement (CaRE ^2^) Health Equity Center, USA

**Keywords:** Acinar ductal metaplasia, Biomechanics, Pancreas plasticity, Pancreatic cancer, Microrheology

## Abstract

The pancreatic ductal adenocarcinoma (PDAC) tumor microenvironment is distinguished by a high degree of fibrosis and inflammation, known as desmoplasia. Desmoplasia increases the stromal deposition and extracellular matrix (ECM) stiffness observed in the tumor microenvironment, contributing to the dampened penetration of pharmacological agents. The molecular and biophysical composition of the ECM during the earliest cellular changes in the development of PDAC, i.e. acinar ductal metaplasia (ADM), has not been extensively explored. We report that the mRNA expression of key protein components of the ECM increases during ADM in *p48^Cre/+^;LSL-Kras^G12D^* (*KC*) mouse acinar organoids cultured in Matrigel. Treatment of the organoids with small molecular weight epigenetic modulating compounds that inhibit or reverse ADM (largazole, FK228 and chaetocin) dramatically reduced the tissue mRNA expression of collagens, hyaluronan synthase, laminin and fibronectin. The storage moduli, determined by video tracking of fluorescent nanoparticles embedded into the Matrigel, increased during ADM and was reduced following treatment with the epigenetic modulating compounds. We report that the ECM of mouse organoids stiffens during ADM and is further enhanced by the presence of mutant *Kras*. Moreover, select HDAC and HMT inhibitors reduced the mRNA expression of ECM components and ECM stiffness during inhibition and reversal of ADM, suggesting that these compounds may be useful as adjuvants to enhance the tumor penetration of agents used to treat PDAC.

## 1. Introduction

Pancreatic ductal adenocarcinoma (PDAC) is the third leading cause of cancer-related deaths in the United States and is predicted to become the second most lethal cancer by 2030 [1,2]. Reasons for the high mortality associated with PDAC include lack of early detection methods and ineffective treatments. Part of PDAC progression includes the formation of a dense and fibrous combination of cellular and non-cellular components called the desmoplastic reaction. Desmoplasia results in the infiltration of fibroblasts, immune cells, and deposition of collagen types I, III, and IV, fibronectin, laminin, and hyaluronan, resulting in dramatic changes in overall tissue heterogeneity and elasticity, as well as accompanying increased interstitial fluid pressure [3]. Chemoresistance in PDAC is contributed in part to the poor tumor penetration and limited perfusion of drugs due to the increased interstitial pressure of the tumor.

Acinar ductal metaplasia (ADM) is a process by which pancreatic acinar cells de-differentiate into ductal-like cells. When combined with genetic mutations, such as the oncogene *Kras*, ADM progresses into pancreatic intraepithelial neoplasia (PanINs) and eventually PDAC [4, 5]. ADM serves as a protective mechanism during periods of pancreatic damage [6-8]. Hallmarks of ADM include loss of zymogen granules, reduced expression of the acinar marker amylase 2a and increased expression of the ductal marker cytokeratin 19. Dedifferentiation of acinar cells to ductal epithelial cells and embryonic progenitor cells with increased proliferative activity are believed to contribute to the regeneration of acinar structures and repopulation of the pancreas [9]. During intense inflammation of the pancreas, cellular remodeling occurs, acinar cells die or de-differentiate into ductal cells and quiescent pancreatic stellate cells become activated and produce extracellular matrix proteins including collagen fibers, leading to fibrosis.

Microrheology has been increasingly used to investigate the local biomechanics of tissues [10,11]. Microbiological techniques, especially passive approaches, which perturb samples non-invasively, have become increasingly popular to probe the mechanics of cancer tissue [12]. In particular, video particle tracking can reveal the spatiotemporal information and heterogeneity of the tumor microenvironment and has been previously used for in vitro PDAC ECM investigations [13,14]. Moreover, these microscale measurements can target precise regions of interest [15,16].

The association between the molecular and biophysical changes to the ECM during pancreatic ADM has not been extensively explored. The intent of this study is to evaluate the mechanical stiffness of the ECM during ADM and following inhibition or reversal of ADM by small molecular weight compounds [17,18].

## 2. Materials and methods

### 2.1. Mice

The *p48^Cre/+^;LSL-Kras^G12D^* (*KC*) line was generated by breeding *p48^Cre/+^ (Cre) and LSL-Kras^G12D/+^* mice. Genotyping was performed through Transnetyx (Cordvoa, TN). All breeding activities and experimental procedures were approved through IACUC protocol 202109058 at the University of Florida.

### 2.2. Chemicals, compounds and reagents

Trichostatin A (TSA) was purchased from Cayman Chemicals (#89730), FK228 from Sigma-Aldrich, (#SML1175) and chaetocin from Cayman Chemicals (#11076). Largazole homodimer was synthesized as previously reported [19]. Growth factor reduced Matrigel was purchased from Corning (#354230).

### 2.3. 3D organoid cultures

Primary mouse pancreatic acinar cells from *Cre* and *KC* mice were cultured in 96 well plates in Matrigel to allow for ADM to occur as previously reported [17,18]. Microscopic counting of the acinar and ductal cells was performed on the cultures of both mouse strains to obtain a percent of ADM (total number of ducts divided by the total number of objects) from triplicate wells.

### 2.4. Video particle tracking

Video particle tracking was performed to obtain the microrheological properties of the ECM. Carboxylated fluorescent (FluoS-pheresTM, F8823, yellow-green, 505/515 nm) polystyrene particles with a diameter of 1 μm (Invitrogen^™^ FluoSpheres^™^, Carboxylate-Modified Microspheres) were PEGylated with methoxypolyethylene glycol amine (PEG-NH2). The probe particles were embedded into the Matrigel and cell suspension (40 μl of Matrigel and cells, 60 μl of complete media with 30 % v/v fluorescent probe particles). The probe particles were recorded using a Nikon, Eclipse Ti-3 inverted microscope with a 20 × objective and a CMOS camera (Nikon, DS-Qi2). The microscope was placed on an inflated air table (constant nitrogen air pressure of 80 psi). Videos were acquired using the respective filters and emissions for the fluorescent channel. The resolution of the video was set to 14-bit 536 × 536 at an autoexposure of 1 ms following autoscaling of the field for visualization of the probe particles. All videos were taken for 10 s, acquiring, on average, 43 frames per second. Particle tracking of the resulting videos was performed using TrackMate ImageJ. Particle coordinates were then exported and analyzed in MATLAB. The overall storage modulus of the ADM tissue were determined using the generalized Stokes-Einstein equation [20,21].

### 2.5. Validation of video particle tracking

The video particle tracking assay was validated by measuring the viscosities of various glycerol solutions at 20 °C. The viscosities determined by video particle tracking were compared to literature values based on the formula developed in Ref. [22]. Fluorescent probe particles with a diameter of 1 μm were used for this evaluation. The storage moduli were determined by video particle tracking in various concentrations of Matrigel and cell culture media in the absence of any cells. These include the Matrigel Media composition that the cells are cultured in; 40 μl Matrigel and 60 μl complete media (1 × ), as well as 0.25 × , 0.5 × and 1.5 × Matrigel.

### 2.6. Compound treatments

For microrheological measurements, ADM inhibition cultures were immediately treated with the compounds on day 0. For ADM reversal, cells underwent two days of ADM, followed by 24 h of drug reversal.

### 2.7. Gene expression analysis

Bulk transcriptomic sequencing data were obtained from our prior publication [17] (GSE236292). RNA was isolated from the Matrigel as previously described [23] and 50 ng of total RNA was converted into cDNA using standard techniques. The qPCR was performed using the QuantStudio 7 Flex Real-Time PCR System (Thermo). Calculations for the $2_{T}^{-\Delta \Delta C}$ equation [24] were obtained using 18S rRNA as the normalizer. Primer sequences are available upon request.

## 3. Results

### 3.1. Modulation of ECM components during ADM

**Fig. 1.**
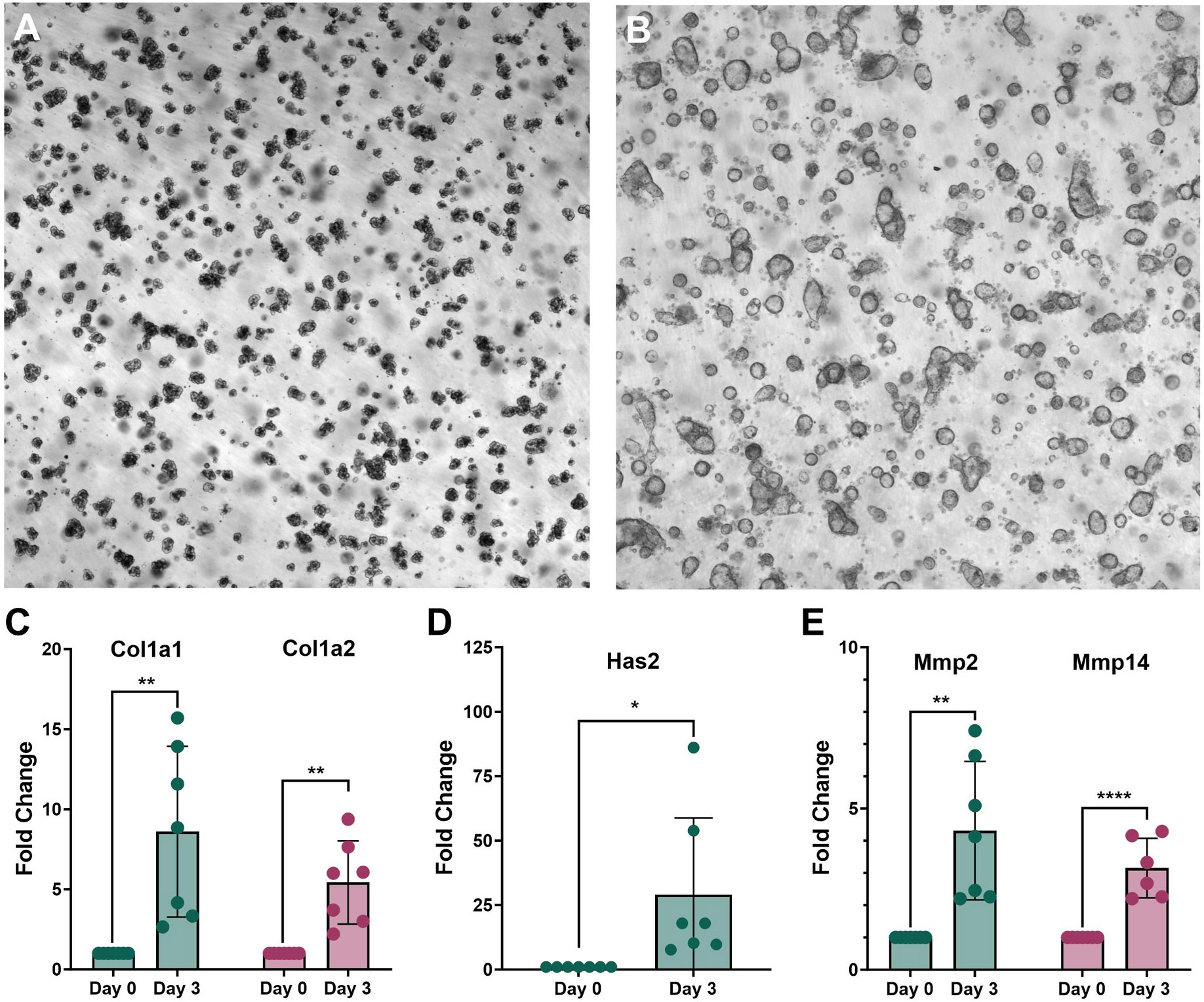
Expression of extracellular matrix components during ADM. Pancreatic acinar cells from *KC* mice underwent ADM over 3 days of culture on Matrigel. Shown are the brightfield images for the (A) Day 0 (primarily acinar cell clusters) and (B) Day 3 (primarily ductal cells) cultures (4 × magnification). The mRNA expression of (C) collagens (D) hyaluronan synthase and (E) matrix metalloproteinases at Day 0 and Day 3 of ADM are presented. The mean data (biological replicates) ± SD are shown. *p < 0.05; **, p < 0.005 and ****, p < 0.0005.

The acinar cells from *KC* mice underwent ADM over 3 days (Fig. 1A and B). The relationship between the expression of components of the ECM and ADM in acinar tissues that contains the *Kras^G12D^* mutation was observed. The mRNA expression of Col1a1 and Col1a2 increased up to 8-fold over the 3-day time course of ADM in *KC* acinar cells, while Has2 mRNA increased by over 20-fold (Fig. 1C-E).

### 3.2. Assay validation

**Fig. 2.**
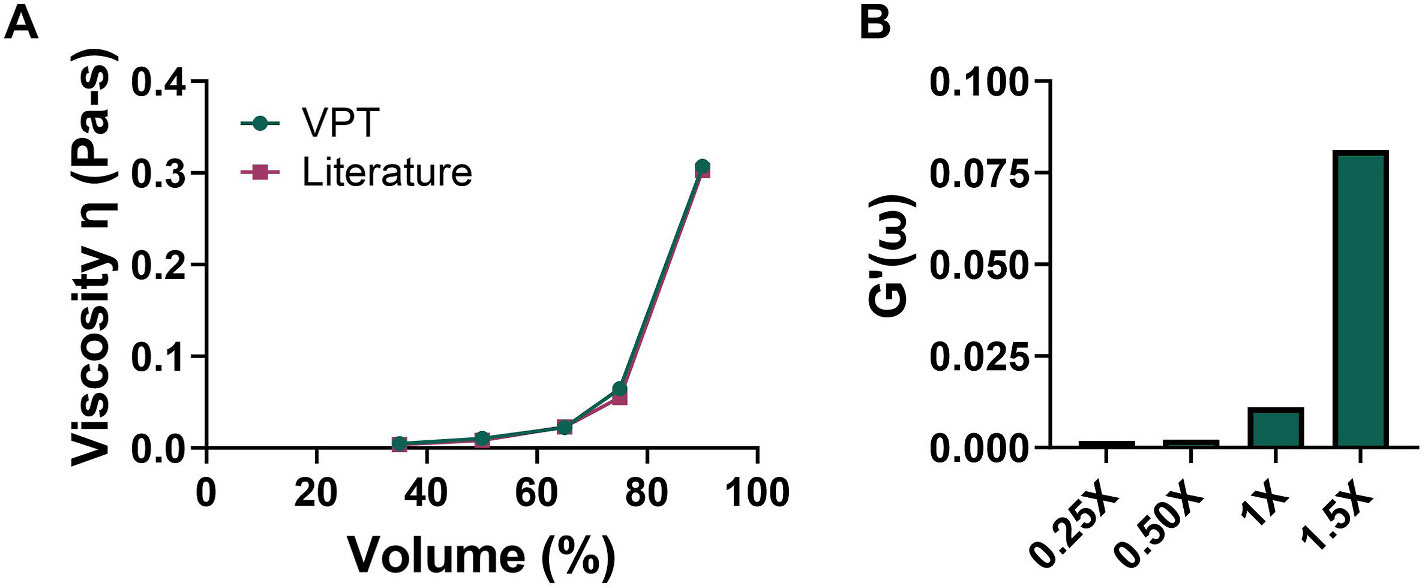
Assay validation for video particle tracking analysis of shear modulus. (A) Viscosity of glycerol solutions as determined by video particle tracking analysis compared to literature values. (B) Storage modulus (reported at 10 Hz) of Matrigel suspension of varying degrees of stiffness using video particle tracking. 1 × represents the Matrigel/media composition used in the cell culture experiments.

Before obtaining microrheological measurements of ADM, the video particle tracking method was validated. This methodology applies microrheological measurements that tracks the Brownian motion of PEGylated fluorescent particles embedded into the Matrigel. The mechanical stiffness of a series of glycerol solutions was determined (Fig. 2A). As an additional validation, a series of cell-free, aqueous dispersions of Matrigel was evaluated for their degree of stiffness. A good correlation exists between the increasing amount of Matrigel in the dispersions and the storage moduli (Fig. 2B).

### 3.3. The ECM stiffens in KC organoids undergoing ADM

**Fig. 3.**
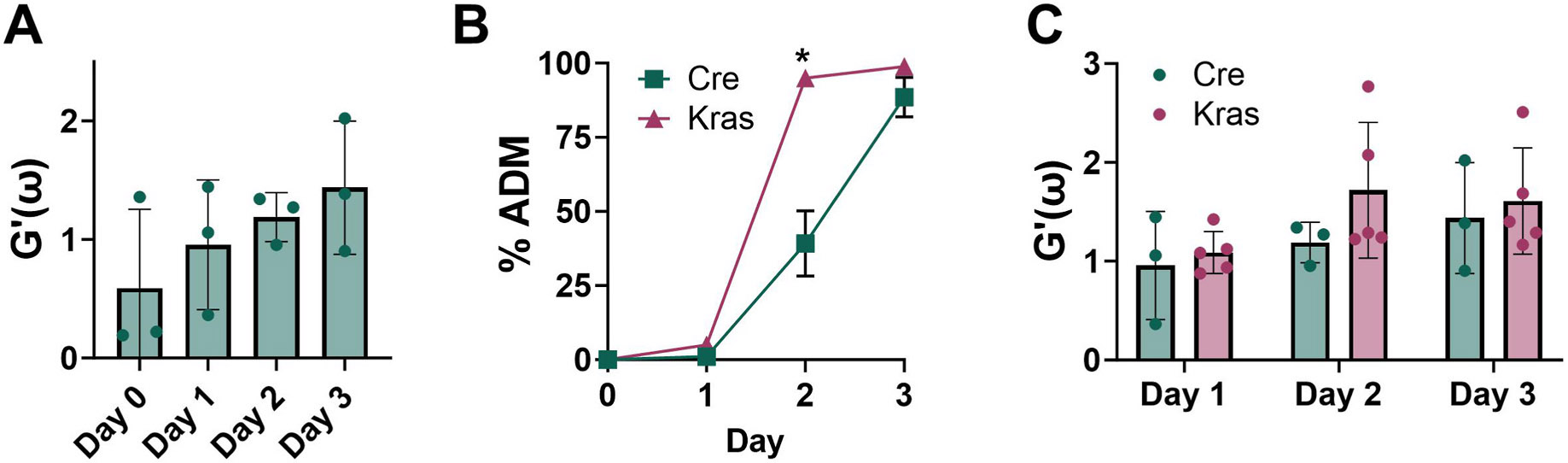
ECM stiffness increases in *KC* mouse organoids undergoing ADM. (A) Storage moduli (reported at 10 Hz) during the progression of ADM for *Cre* mouse organoids. (B) Microscopic counting of acinar and ductal cells to obtain the percent of ADM for *Cre* (n = 3) and *KC* (n = 3) ADM organoids. (C) Storage moduli (reported at 10 Hz) during ADM progression for *Cre* and *KC* mouse organoids. Each data point is from an independent replicate; mean data (biological replicates) ± SD are shown. *p < 0.05.

Next, we used video particle tracking analysis to measure the stiffness of the ECM during in vitro ADM. A trend of increasing storage moduli with days of ADM culture was observed in the *Cre* mouse organoid cultures (Fig. 3A). Similar to our prior report [18], the degree of ADM was enhanced for the *KC* cultures compared to the *Cre* control (Fig. 3B). The storage moduli increased in the *KC* organoids compared to the *Cre* controls (Fig. 3C). We conclude that the storage modulus of the ECM increases during ADM and the presence of the *Kras^G12D^* mutation enhances the ECM stiffness.

### 3.4. Epigenetic modulating compounds reduce the storage moduli in KC organoids undergoing ADM

**Fig. 4.**
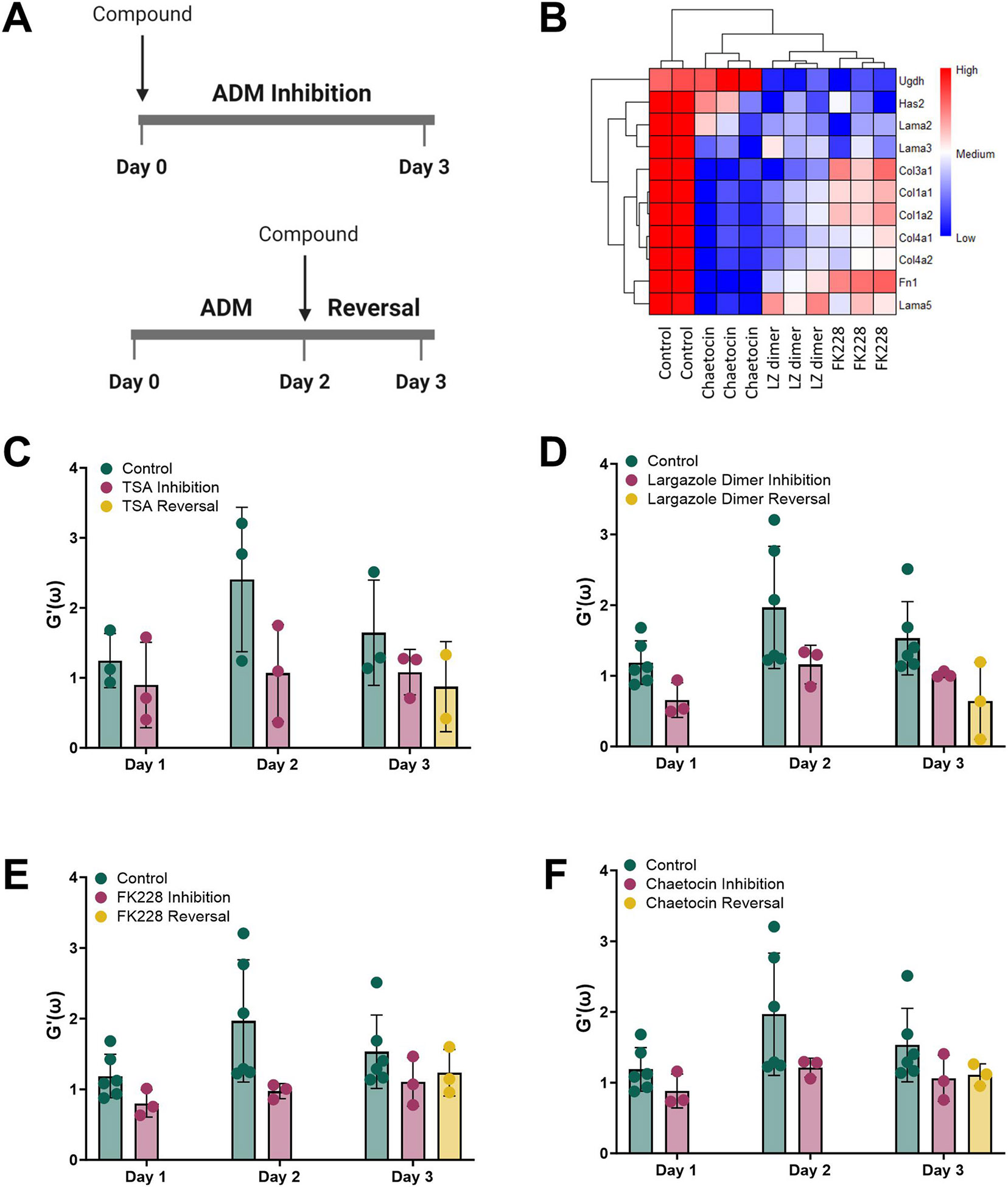
Gene expression and ADM storage moduli changes by epigenetic compound treatment. (A) ADM inhibition and reversal treatment schema in *KC* mouse organoid cultures. (B) Heatmap of the mRNA expression of ECM-related genes as determined by RNA sequencing in *KC* cultures treated with 1 μM chaetocin, 10 μM largazole (LZ) dimer and 1 μM FK228 following treatment in the ADM reversal mode. Storage moduli (reported at 10 Hz) of *KC* mouse organoids during ADM inhibition and reversal modes following treatment of (C) 1 μM TSA, (D) 10 μM largazole dimer, (E) 1 μM FK228 and (F) 1 μM chaetocin. The mean data (biological replicates) ± SD are shown.

We asked if epigenetic modulating compounds discovered in our medium throughput screen [17] could alter the tissue mRNA expression of components of the ECM (Fig. 4A). These include the HDAC inhibitors (TSA, largazole homodimer and FK228) and the HMT inhibitor chaetocin. Largazole homodimer prodrug was designed to have improved stability while also liberating two equivalents of active species, largazole thiol [25]. The acinar cultures were treated with these four compounds at concentrations that were shown to be nontoxic in our prior study [17]. The mRNA expression of collagens, laminins, fibronectin and hyaluronan synthase were significantly reduced following ADM reversal with compounds largazole dimer, FK228 and chaetocin (Fig. 4B). Next, we sought to examine the relationship between the ECM stiffness and ADM inhibition and reversal with the epigenetic modulating compounds. The storage moduli were measured during ADM in the presence of the four epigenetic modulating compounds. All four compounds reduced the storage modulus in both ADM inhibition and ADM reversal modes compared to control, however the differences are not statistically significant (Fig. 4C-F). We conclude that select HDAC and HMT inhibitors can reduce the stiffening of the ECM during ADM inhibition and following ADM reversal in mouse organoids with mutant *Kras*.

## 4. Discussion

While ADM within the invasive front of PDAC is associated with desmoplasia [26] and regions of ADM have been characterized by the deposition of the ECM component hyaluronan [4], the relationship between ECM stiffening and ADM has not been described to our knowledge. We applied video particle tracking analysis to quantify the storage moduli of the ECM during in vitro ADM of acinar tissue from control and *KC* mice. The storage moduli increased during 3 days of ADM in both *Cre* and *KC* mice; a slight increase in stiffening of the ECM was observed in *KC* compared to *Cre* mice, however the differences were not statistically significant (Fig. 3C). The tissue mRNA expression of major components of the desmoplastic reaction including collagens, hyaluronan synthase and matrix metalloproteinases dramatically increased during ADM (Fig. 1).

Quiescent pancreatic stellate cells (PSCs) represent about 4 % of the cells in the normal pancreas [27] and can transform into myofibroblast-like activated PSCs during development of diseases such as PDAC or chronic pancreatitis [28]. Activated PSCs have elevated migration and proliferative ability and produce large amounts of ECM-remodeling proteins such as collagens, laminin, hyaluronic acid and fibronectin [28]. PSC activation and ADM occurred simultaneously during caerulein-induced pancreatitis in mice [29]. Our study applied low speed centrifugation to purify the acinar cell clusters, therefore it is likely that some quiescent PSCs were present in the cultures along with the acini. Transformation of quiescent to activated PSCs likely occurred during ADM and is responsible for the increased expression of ECM-related remodeling genes (Fig. 1).

Increased structural integrity of the pancreatic tissue due to large amounts of hyaluronan and other ECM proteins contributes to reduced drug penetration in PDAC. Moreover, the barrier created by the ECM proteins impedes the penetration of immune cells to the tumor. Pharmacological modulation of the degree of ECM stiffness has the potential for improving the therapeutic efficacy for PDAC. In fact, mechanotherapeutics targeting cell-ECM signaling pathways are being explored in PDAC [30]. Prior attempts to reduce the ECM barrier with pegylated recombinant hyaluronidase (PEGPH20), enhanced gemcitabine activity in preclinical, mouse studies [31], however, this therapeutic approach failed in a Phase I/II randomized trial due to enhanced toxicity of the FOLFIRINOX plus PEGPH20 arm [32].

It is noteworthy that small molecular weight epigenetic modulators, previously shown by our group to inhibit and reverse ADM [17], significantly reduced the mRNA expression of ECM-related proteins such as hyaluronidase, collagens, laminin and fibronectin (Fig. 4B). Treating the *KC* ADM cultures with the small molecular weight compounds largazole, FK228 and chaetocin reduced the degree of stiffness at Day 3 of ADM (Fig. 4). Thus, these epigenetic regulators not only reduced the mRNA expression of proteins that are present in the ECM, but also reduced ECM stiffness. Given that the mouse tissues studied here contain mutant *Kras*, these findings may be applicable to human PDAC, where over 90 % of cases harbor the *KRAS* mutation. As FK228 (depsipeptide) is an FDA approved class I HDAC inhibitor used to treat cutaneous T-cell lymphoma [33], studies combining HDAC inhibitors with standard of care treatments for PDAC are warranted and may result in enhanced tumor penetration with reduced toxicity compared to other therapeutic modalities.

## Abbreviations:

ADM: acinar ductal metaplasia
CPM: count per million
HDAC: histone deacetylase
HMT: histone methyltransferase
PDAC: pancreatic ductal adenocarcinoma
ECM: extracellular matrix
TSA: trichostatin A
PSC: pancreatic stellate cells
